# 3-Oxapentane-1,5-diyl dicarbamate

**DOI:** 10.1107/S1600536812011981

**Published:** 2012-03-24

**Authors:** Zhi Li

**Affiliations:** a Department of Chemistry, School of Science, Beijing Jiaotong University, Beijing 100044, People’s Republic of China

## Abstract

The complete mol­ecule of the title compound, C_6_H_12_N_2_O_5_, is generated by a rotation about a twofold axis. The conformation along the bond sequence linking the two amino groups is *trans*-*trans*-(+)*gauche*-*trans*-*trans*. In the crystal, N—H⋯O hydrogen bonds link the mol­ecules into a three-dimensional supra­molecular architecture.

## Related literature

For self-assembled mono-layers of alkyl carbamate and alkyl dicarbamate, see: Kim *et al.* (2003 ▶, 2005*a* ▶,*b* ▶). For the synthesis of the title compound, see: Sidney *et al.* (1965 ▶); Takeuchi & Ninagawa (1971 ▶); Takeuchi (1974 ▶). For a closely related structure and background references, see: Xia *et al.* (2010 ▶, 2011 ▶).
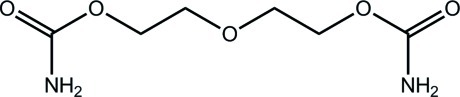


## Experimental

### 

#### Crystal data

C_6_H_12_N_2_O_5_
*M* *_r_* = 192.18
Monoclinic, 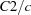

*a* = 14.263 (4) Å
*b* = 5.1412 (15) Å
*c* = 12.276 (4) Å
β = 99.393 (5)°
*V* = 888.1 (5) Å^3^
*Z* = 4
Mo *K*α radiation
μ = 0.13 mm^−1^
*T* = 294 K
0.30 × 0.20 × 0.14 mm

#### Data collection

Bruker SMART CCD area-detector diffractometer
Absorption correction: multi-scan (*SADABS*; Sheldrick, 1996 ▶) *T*_min_ = 0.960, *T*_max_ = 0.983
2358 measured reflections
904 independent reflections
748 reflections with *I* > 2σ(*I*)
*R*_int_ = 0.019

#### Refinement

*R*[*F* ^2^ > 2σ(*F* ^2^)] = 0.031
*wR*(*F* ^2^) = 0.084
*S* = 1.06
904 reflections
69 parameters
H atoms treated by a mixture of independent and constrained refinement
Δρ_max_ = 0.16 e Å^−3^
Δρ_min_ = −0.14 e Å^−3^

### 

Data collection: *SMART* (Bruker, 2007 ▶); cell refinement: *SAINT* (Bruker, 2007 ▶); data reduction: *SAINT*; program(s) used to solve structure: *SHELXS97* (Sheldrick, 2008 ▶); program(s) used to refine structure: *SHELXL97* (Sheldrick, 2008 ▶); molecular graphics: *SHELXTL* (Sheldrick, 2008 ▶); software used to prepare material for publication: *SHELXTL*.

## Supplementary Material

Crystal structure: contains datablock(s) global, I. DOI: 10.1107/S1600536812011981/tk5072sup1.cif

Structure factors: contains datablock(s) I. DOI: 10.1107/S1600536812011981/tk5072Isup2.hkl

Supplementary material file. DOI: 10.1107/S1600536812011981/tk5072Isup3.cml

Additional supplementary materials:  crystallographic information; 3D view; checkCIF report

## Figures and Tables

**Table 1:** Hydrogen-bond geometry (Å, °)

*D*—H⋯*A*	*D*—H	H⋯*A*	*D*⋯*A*	*D*—H⋯*A*
N1—H1*A*⋯O1^i^	0.872 (18)	2.046 (18)	2.9086 (17)	169.9 (14)
N1—H1*B*⋯O2^ii^	0.852 (17)	2.381 (17)	3.1763 (17)	155.6 (14)

Symmetry codes: (i) 
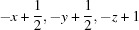
; (ii) 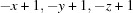.

**Fig. 1.**
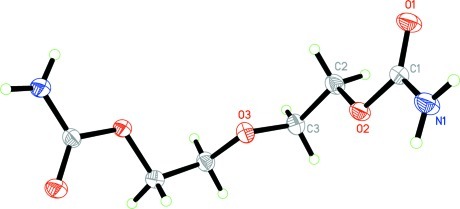
The molecular structure of the title compound, with displacement ellipsoids at the 30% probability level. The molecule has crystallographic twofold symmetry. Unlabelled atoms are related by the symmetry operation 1-x, y, 1/2-z.

**Fig. 2.**
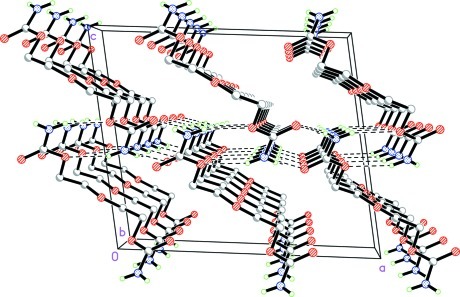
Crystal packing in the title compound where molecules are linked via N–H···O hydrogen bonds (dashed lines). Except for those involved in hydrogen-bonding interactions, H atoms have been omitted for clarity.

## supplementary crystallographic information

### Comment

Recently, self-assembled mono-layers of alkyl carbamates and alkyl dicarbamates have been investigated and characterized (Kim *et al.*, 2003, 2005*a*, 2005*b*). Further, ligands with two amino moieties demonstrate versatile bonding modes to metal ions and readily form coordination polymers or supramolecular compounds (Xia *et al.*, 2010, 2011). For example, 3,3'-(oxybis(ethane-2,1-diyloxycarbonylimino))dipyridinium functions as a ligand as seen in its copper(II) and zinc(II) complexes (Xia *et al.,* 2011). To further investigate this family of ligands and the self-assembled activity of the dicarbamate linked by an ether chain, the title compound, (I), was synthesized and its structure was confirmed by X-ray diffraction.

The title compound contains one half-molecule as it is disposed about a crystallographic twofold axis with the O3 atom lying on the axis (Fig. 1). The conformation along the bond sequence linking the two amino groups is *trans*-*trans*-(+)*gauche*-*trans*-*trans*. The relevant torsion angle are: N1–C1–O2–C2, -178.66 (11)°; C1–O2–C2–C3, -177.18 (10)°; O2–C2–C3–O3, 68.67 (13)°; C2–C3–O3–C3, 170.41 (12)°; C3^i^–O3–C3–C2, 170.41 (12)°, symmetry code (i): 1-*x*, *y*, 1/2-*z*]. In the crystal packing, pairs of intermolecular N–H···O hydrogen bonds link the molecules into a three-dimensional supramolecular architecture (Fig. 2 and Table 1).

### Experimental

The title compound was synthesized by transesterification of ethyl carbamate with 2,2'-oxydiethanol (Sidney *et al.*, 1965; Takeuchi & Ninagawa 1971; Takeuchi 1974) as follows. A solution of ethyl carbamate (8.9 g, 100 mmol) and 2,2'-oxydiethanol (1.0 g, 10 mmol) in toluene (30 ml) was heated to reflux in the presence of a catalytic amount of ZnCl_2_ for 8 h. After cooling to room temperature, the solvent was evaporated under vacuum. The residue was subjected to flash chromatography and the title compound was obtained as colourless crystals (0.97 g; Yield: 50%; *M*.pt: 428–429 K). Crystals were grown by slow evaporation from its DMF solution.

### Refinement

Carbon-bound H-atoms were placed in calculated positions (C—H = 0.97 Å) and were included in the refinement in the riding model approximation, with *U*_iso_(H) set to 1.2*U*_eq_(C). The amino group H-atoms were located in a difference Fourier map, and were refined freely.

### Crystal data

**Table:**

C_6_H_12_N_2_O_5_	*F*(000) = 408
*M**_r_* = 192.18	*D*_x_ = 1.437 Mg m^−^^3^
Monoclinic, *C*2/*c*	Melting point: 428 K
Hall symbol: -C 2yc	Mo *K*α radiation, λ = 0.71073 Å
*a* = 14.263 (4) Å	Cell parameters from 1304 reflections
*b* = 5.1412 (15) Å	θ = 3.4–26.0°
*c* = 12.276 (4) Å	µ = 0.13 mm^−^^1^
β = 99.393 (5)°	*T* = 294 K
*V* = 888.1 (5) Å^3^	Plate, colourless
*Z* = 4	0.30 × 0.20 × 0.14 mm

### Data collection

**Table:**

Bruker SMART CCD area-detector diffractometer	904 independent reflections
Radiation source: fine-focus sealed tube	748 reflections with *I* > 2σ(*I*)
Graphite monochromator	*R*_int_ = 0.019
φ and ω scans	θ_max_ = 26.3°, θ_min_ = 2.9°
Absorption correction: multi-scan (*SADABS*; Sheldrick, 1996)	*h* = −16→17
*T*_min_ = 0.960, *T*_max_ = 0.983	*k* = −6→6
2358 measured reflections	*l* = −8→15

### Refinement

**Table:**

Refinement on *F*^2^	Secondary atom site location: difference Fourier map
Least-squares matrix: full	Hydrogen site location: inferred from neighbouring sites
*R*[*F*^2^ > 2σ(*F*^2^)] = 0.031	H atoms treated by a mixture of independent and constrained refinement
*wR*(*F*^2^) = 0.084	*w* = 1/[σ^2^(*F*_o_^2^) + (0.0407*P*)^2^ + 0.3363*P*] where *P* = (*F*_o_^2^ + 2*F*_c_^2^)/3
*S* = 1.06	(Δ/σ)_max_ < 0.001
904 reflections	Δρ_max_ = 0.16 e Å^−^^3^
69 parameters	Δρ_min_ = −0.14 e Å^−^^3^
0 restraints	Extinction correction: *SHELXL97* (Sheldrick, 2008), Fc^*^=kFc[1+0.001xFc^2^λ^3^/sin(2θ)]^-1/4^
Primary atom site location: structure-invariant direct methods	Extinction coefficient: 0.029 (3)

### Special details

**Table:**

Geometry. All e.s.d.'s (except the e.s.d. in the dihedral angle between two l.s. planes) are estimated using the full covariance matrix. The cell e.s.d.'s are taken into account individually in the estimation of e.s.d.'s in distances, angles and torsion angles; correlations between e.s.d.'s in cell parameters are only used when they are defined by crystal symmetry. An approximate (isotropic) treatment of cell e.s.d.'s is used for estimating e.s.d.'s involving l.s. planes.
Refinement. Refinement of *F*^2^ against ALL reflections. The weighted *R*-factor *wR* and goodness of fit *S* are based on *F*^2^, conventional *R*-factors *R* are based on *F*, with *F* set to zero for negative *F*^2^. The threshold expression of *F*^2^ > σ(*F*^2^) is used only for calculating *R*-factors(gt) *etc*. and is not relevant to the choice of reflections for refinement. *R*-factors based on *F*^2^ are statistically about twice as large as those based on *F*, and *R*- factors based on ALL data will be even larger.

### Fractional atomic coordinates and isotropic or equivalent isotropic displacement parameters (Å^2^)

**Table:**

	*x*	*y*	*z*	*U*_iso_*/*U*_eq_	
O1	0.25476 (6)	0.5124 (2)	0.40946 (8)	0.0461 (3)	
O2	0.39925 (5)	0.69845 (18)	0.42131 (7)	0.0374 (3)	
O3	0.5000	0.8767 (2)	0.2500	0.0358 (3)	
N1	0.37706 (8)	0.3841 (3)	0.53985 (10)	0.0451 (3)	
H1A	0.3429 (12)	0.260 (3)	0.5619 (13)	0.054 (4)*	
H1B	0.4360 (12)	0.398 (3)	0.5657 (13)	0.051 (4)*	
C1	0.33731 (8)	0.5279 (2)	0.45456 (10)	0.0333 (3)	
C2	0.35837 (8)	0.8634 (3)	0.33006 (11)	0.0392 (3)	
H2A	0.3086	0.9708	0.3518	0.047*	
H2B	0.3306	0.7572	0.2678	0.047*	
C3	0.43501 (9)	1.0321 (3)	0.29804 (11)	0.0379 (3)	
H3A	0.4071	1.1628	0.2455	0.045*	
H3B	0.4683	1.1206	0.3628	0.045*	

### Atomic displacement parameters (Å^2^)

**Table:**

	*U*^11^	*U*^22^	*U*^33^	*U*^12^	*U*^13^	*U*^23^
O1	0.0300 (5)	0.0575 (6)	0.0477 (6)	−0.0091 (4)	−0.0029 (4)	0.0107 (5)
O2	0.0266 (4)	0.0479 (5)	0.0365 (5)	−0.0039 (4)	0.0020 (4)	0.0091 (4)
O3	0.0363 (6)	0.0328 (6)	0.0403 (7)	0.000	0.0128 (5)	0.000
N1	0.0301 (6)	0.0570 (8)	0.0464 (7)	−0.0046 (5)	0.0007 (5)	0.0168 (6)
C1	0.0277 (6)	0.0402 (7)	0.0321 (6)	−0.0025 (5)	0.0055 (5)	−0.0009 (5)
C2	0.0309 (6)	0.0480 (8)	0.0386 (7)	0.0031 (5)	0.0050 (5)	0.0089 (6)
C3	0.0376 (7)	0.0365 (7)	0.0414 (7)	0.0034 (5)	0.0119 (6)	0.0027 (5)

### Geometric parameters (Å, º)

**Table:**

O1—C1	1.2191 (15)	N1—H1B	0.852 (17)
O2—C1	1.3543 (15)	C2—C3	1.4973 (18)
O2—C2	1.4494 (15)	C2—H2A	0.9700
O3—C3	1.4228 (14)	C2—H2B	0.9700
O3—C3^i^	1.4228 (14)	C3—H3A	0.9700
N1—C1	1.3305 (17)	C3—H3B	0.9700
N1—H1A	0.872 (18)		
			
C1—O2—C2	114.33 (9)	C3—C2—H2A	109.9
C3—O3—C3^i^	111.63 (13)	O2—C2—H2B	109.9
C1—N1—H1A	117.6 (10)	C3—C2—H2B	109.9
C1—N1—H1B	121.0 (11)	H2A—C2—H2B	108.3
H1A—N1—H1B	120.9 (15)	O3—C3—C2	109.65 (11)
O1—C1—N1	125.25 (12)	O3—C3—H3A	109.7
O1—C1—O2	122.31 (11)	C2—C3—H3A	109.7
N1—C1—O2	112.44 (11)	O3—C3—H3B	109.7
O2—C2—C3	108.85 (10)	C2—C3—H3B	109.7
O2—C2—H2A	109.9	H3A—C3—H3B	108.2
			
C2—O2—C1—O1	1.42 (17)	C3^i^—O3—C3—C2	170.41 (12)
C2—O2—C1—N1	−178.66 (11)	O2—C2—C3—O3	68.67 (13)
C1—O2—C2—C3	−177.18 (10)		

Symmetry code: (i) −*x*+1, *y*, −*z*+1/2.

### Hydrogen-bond geometry (Å, º)

**Table:**

*D*—H···*A*	*D*—H	H···*A*	*D*···*A*	*D*—H···*A*
N1—H1*A*···O1^ii^	0.872 (18)	2.046 (18)	2.9086 (17)	169.9 (14)
N1—H1*B*···O2^iii^	0.852 (17)	2.381 (17)	3.1763 (17)	155.6 (14)

Symmetry codes: (ii) −*x*+1/2, −*y*+1/2, −*z*+1; (iii) −*x*+1, −*y*+1, −*z*+1.
